# Follicular development of fetal gonads under the skin of adult mice

**DOI:** 10.1093/lifemedi/lnaf007

**Published:** 2025-02-24

**Authors:** Jiyu Chen, Chang Liu, Yongqin Yu, Xiaoying Ye, Lin Liu, Zhengmao Zhu

**Affiliations:** Department of Genetics and Cell Biology, College of Life Science, Nankai University, Tianjin 300071, China; State Key Laboratory of Medicinal Chemical Biology, Nankai University, Tianjin 300350, China; Department of Genetics and Cell Biology, College of Life Science, Nankai University, Tianjin 300071, China; State Key Laboratory of Medicinal Chemical Biology, Nankai University, Tianjin 300350, China; Department of Genetics and Cell Biology, College of Life Science, Nankai University, Tianjin 300071, China; State Key Laboratory of Medicinal Chemical Biology, Nankai University, Tianjin 300350, China; Department of Genetics and Cell Biology, College of Life Science, Nankai University, Tianjin 300071, China; State Key Laboratory of Medicinal Chemical Biology, Nankai University, Tianjin 300350, China; Department of Genetics and Cell Biology, College of Life Science, Nankai University, Tianjin 300071, China; State Key Laboratory of Medicinal Chemical Biology, Nankai University, Tianjin 300350, China; Haihe Laboratory of Cell Ecosystem, Chinese Academy of Medical Sciences & Peking Union Medical College, Tianjin 300020, China; Department of Genetics and Cell Biology, College of Life Science, Nankai University, Tianjin 300071, China; State Key Laboratory of Medicinal Chemical Biology, Nankai University, Tianjin 300350, China; Haihe Laboratory of Cell Ecosystem, Chinese Academy of Medical Sciences & Peking Union Medical College, Tianjin 300020, China

**Keywords:** follicle development, meiosis, gonad, ovary, subcutaneous transplantation

## Abstract

Adult ovarian tissues or biopsies isolated from patients prior to chemotherapy or irradiation can reconstitute ovarian functions when transplanted either in the abdomen or subcutaneously. Subcutaneously transplantation avoids invasive surgery and potential risks associated with internal procedures. We investigated whether functional ovaries could develop subcutaneously from early E12.5 fetal gonads without entering meiosis in mouse model. Unexpectedly, the subcutaneously transplanted fetal gonads failed to undergo folliculogenesis in the recipient mice. The transplanted gonads experienced meiotic deficiency and exhibited significant defects in DNA repair and recombination, increased apoptosis levels. Meiotic defects in the subcutaneous grafts were partly attributable to variations in temperature and oxygen concentration. However, completion of meiotic prophase I was effectively achieved through *in vitro* culture of the gonads at 37°C. Subsequently, the *in vitro* cultured E12.5 gonads, following subcutaneous transplantation, became competent in folliculogenesis, restoring endocrine functions. This finding may have implications for rejuvenating ovarioids from fetal gonad-like cells using pluripotent stem cell technologies, as well as for enhancing endocrine recovery and health span.

## Introduction

Better lifestyles and improved healthcare have significantly increased human life expectancy from 45 to 85 years over the past 150 years [1], but the age at natural menopause has remained approximately 50 years [2]. As a consequence of this extended longevity, many women spend about 40% of their lives in menopause with declined health span, as shown by increased risk of chronic diseases, such as endocrine dysfunction, bone mineral density, cardiovascular disease, and Alzheimer’s disease [3–5]. Frozen-thawed ovarian transplants in women diagnosed with primary ovarian insufficiency can restore long-term ovarian endocrine function, potentially lasting more than 5 years [5–8]. Moreover, the graft sites can be heterotopic (subcutaneous sites, forearm, abdominal wall, and kidney capsule), as the primary objective is not fertility restoration [9–13]. Therefore, we suggest that ovarian tissue frozen at a young age has the potential to restore sex steroid hormone secretion after natural menopause and alleviate menopause-related conditions in aging mammalian females.

Ovarian development requires the interaction between germ cells and various types of somatic cells within follicle structures, which provide essential components and signals for critical process in oogenesis, including meiosis and oocyte growth. Functional oocytes can develop in recipients following the transplantation or grafting of fetal gonads or primordial germ cells (PGCs) aggregated with fetal somatic pre-granulosa cells into the kidney capsule (KC) and ovarian bursa in a mouse model [14–19]. Furthermore, mTOR inhibition by INK128 enhances and prolongs the reconstituted ovarian and endocrine functions in mice experiencing reproductive aging and premature aging [20]. However, the success of the reconstituted ovary (rOvary) is heavily reliant on the somatic cell environment provided by embryonic ovarian tissue. Hayashi et al. established a system in mice that facilitates the differentiation of mouse embryonic stem cells into primordial germ cell-like cells (PGCLCs) and fetal ovarian somatic cell-like cells, subsequently aggregating these cell types to create a rOvary. These reconstituted functional follicle structures are fully capable of supporting oocyte production [21]. A previous study illustrated that mouse fetal ovarian somatic cells facilitate the development of human PGCLCs to the oogonia stage [22]. Recently, Church et al. reported that the simultaneous overexpression of two transcription factors can guide the differentiation of human induced pluripotent stem cells to granulosa-like cells, which can then be aggregated with human PGCLCs. These cells formed ovary-like organoids (ovarioids) and supported human PGCLC development from the promigratory to the gonadal stage [23]. Saitou et al. demonstrated *ex vivo* reconstitution of fetal oocyte development in both humans and cynomolgus monkeys. Through optimized culture of fetal ovary reaggregates over three months, human and monkey oogonia enter and complete the first meiotic prophase, differentiating into diplotene oocytes that form primordial follicles [24]. Evidently, the ovarioid, followed by implantation upon a woman reaching menopause, may represent a potential anti-aging therapy following normal menopause in the future.

If the goal is to restore ovarian function rather than fertility, subcutaneous transplantation may serve as an easy and effective method for ovarioid implantation. Although transplanted mouse rOvaries in the kidney capsule could produce functional oocytes [14, 16, 25–27], it remains unclear whether subcutaneously (SC) transplanted mouse fetal gonads or ovarioids can initiate neo-oogenesis. In this study, we observed that meiosis abnormalities in SC-transplanted fetal embryonic day 12.5 (E12.5) gonads resulted in unsuccessful folliculogenesis. However, these meiosis defects can be alleviated through short-term *in vitro* culture of the fetal gonads. Furthermore, the SC-transplanted cultured fetal gonads by short-term culture *in vitro* successfully achieved folliculogenesis and restored ovarian endocrine function.

## Results

### E12.5 gonads under subcutaneous transplantation fail in folliculogenesis

Current progress has illustrated that granulosa-like cells support germ cell development within ovarioids *in vitro* [21, 23]. Given that kidney capsule (KC)-transplanted mouse fetal gonads or ovarioids enabled the production of functional oocytes [14, 16, 26, 27], we initiated an investigation into the folliculogenesis and development potential of SC-transplanted E12.5 gonads from C57BL/6N mice expressing Actin-GFP. For comparison, we followed previously established protocols [14, 16] to culture intact E12.5 gonads *in vitro* and to transplant E12.5 gonads into the kidney capsule.

**Figure 1.**
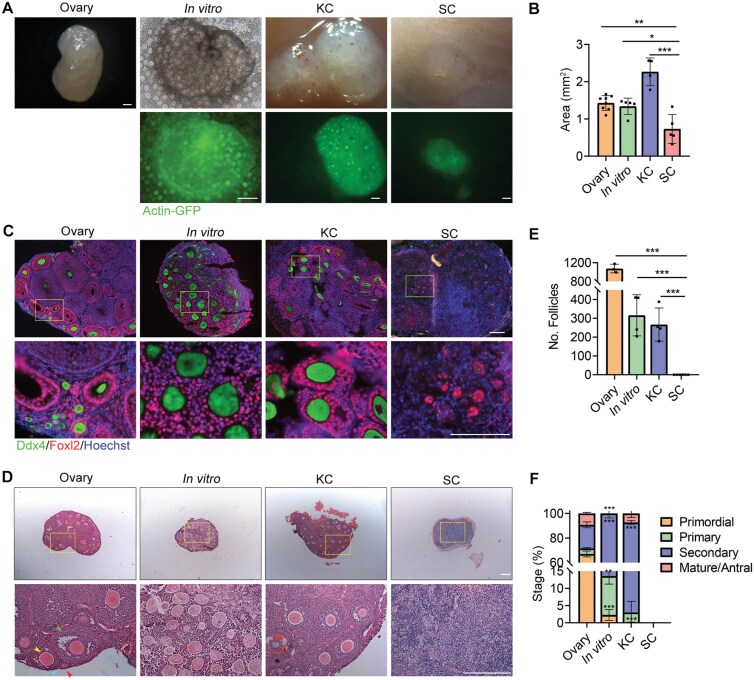
**Inability of E12.5 gonads to form follicular structures following subcutaneous transplantation.**(A) Morphology of a 3-week-old ovary, a gonad cultured *in vitro* for 25 days (*in vitro*), a 25-day transplantation in KC, and a 25-day graft transplanted beneath the skin (SC). Scale bar: 200 μm. (B) Area measurements of the 3-week-old ovary, 25-day *in vitro* cultured gonad, KC graft and SC graft. *n* ≥ 4. Mean ± SD. (C) Immunofluorescence staining of Ddx4 (green) and Foxl2 (red) in 3-week-old ovaries, 25-day *in vitro* cultured gonads, KC grafts and SC grafts. Scale bar: 100 µm. (D) Three-week-old ovaries, *in vitro* cultured gonads, KC grafts and SC grafts were embedded in paraffin, and 8-μm-thick sections were prepared and stained with H&E. Scale bar: 200 µm. (E) The numbers of follicles per 3-week-old ovary, *in vitro* cultured gonad, KC grafts and SC grafts were counted. Combining the counts from every fifth section over the entire tissue yielded the total number of follicles in the tissue. *n* ≥ 3. Mean ± SD. (F) Percentage of different types of follicles per sample. *n* ≥ 3. Mean ± SD. *P < 0.05, **P < 0.01, ***P < 0.001, ns = not significant. Tukey’s multiple comparisons test for (B), (E), (F).

Twenty-five days post-transplantation, we observed that SC-transplanted gonads were significantly smaller than the KC-implanted gonads, *in vitro*-grown gonads, and the ovaries from three-week-old mice (Fig. 1A and 1B). Immunofluorescence staining for Ddx4, a hallmark of primordial and gonadal germ cells [28, 29], and Foxl2, a pre-granulosa cell marker [30], revealed an absence of Ddx4-positive cells in the SC-transplanted gonads, whereas Foxl2-positive cells were present (Fig. 1C). We then examined the histological differences among the SC-implanted grafts, KC-transplanted gonads, *in vitro*-cultured gonads, and 3-week-old mouse ovaries. Both KC-implanted grafts and *in vitro*-grown gonads contained follicles at all stages of folliculogenesis and appeared similar to those from the ovaries of 3-week-old mice (Fig. 1D). Consistent with the immunofluorescence staining results for Ddx4 and Foxl2 (Fig. 1C), we observed a lack of follicle structures in the SC-implanted grafts at day 25 post-transplantation (Fig. 1D and 1E). Furthermore, both *in vitro* cultured and KC-implanted gonads exhibited significantly fewer follicles compared to the ovaries of three-week-old mice (Fig. 1E). Additionally, the ratios of secondary follicles increased in both the *in vitro* and KC-implanted groups (Fig. 1F). At both 16 days and 8 weeks following transplantation, SC grafts also lacked follicular structures (Fig. S1). Therefore, the data suggest that E12.5 gonads transplanted subcutaneously do not successfully develop follicular structures.

### Transplanted fetal E12.5 gonads situated subcutaneously exhibited meiotic defects

**Figure 2.**
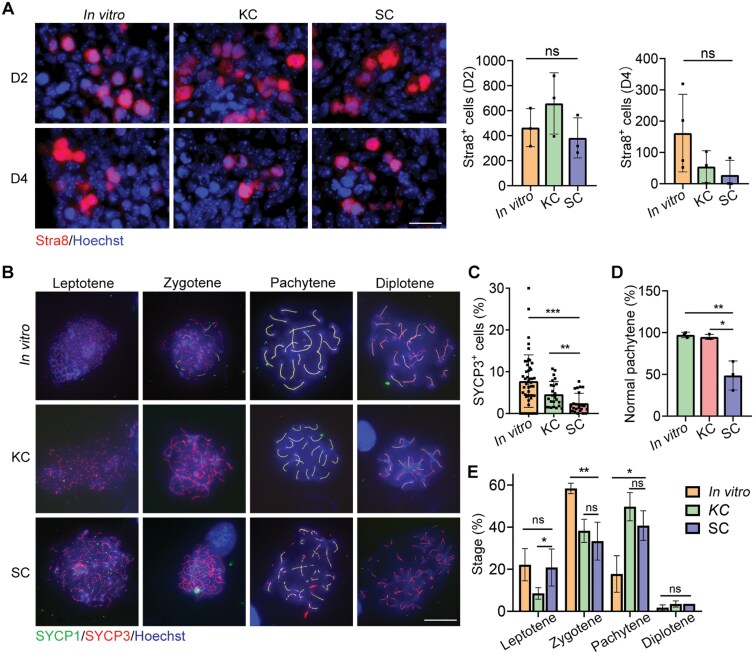
**Meiotic defects in subcutaneous grafts.**(A) Immunofluorescence staining of Stra8 (red) of the 2- and 4-day *in vitro* cultured gonad, KC graft and SC graft transplanted for two and four days. Scale bar: 20 µm. The number of Stra8-positive cells in *in vitro* cultured gonads, KC grafts and SC grafts was counted. *n* ≥ 3. Mean ± SD. (B) Meiocyte spread and immunofluorescence staining of SYCP1 (green) and SYCP3 (red) in E16.5 female gonads, *in vitro* cultured gonads, KC transplants and SC transplants at 4 days. Scale bar: 20 μm. (C) Percentage of SYCP3^+^ cells in E16.5 female gonads, *in vitro* cultured gonads, a 4-day KC transplant and SC transplant. Mean ± SD. (D) Percentage of normal number of synaptonemal complexes at pachytene in gonad cultured *in vitro*, a 4-day KC graft and SC graft. Mean ± SD. (E) Percentage of different developmental stages of meiotic cells in cultured gonads and grafts. *P < 0.05, **P < 0.01, ***P < 0.001, ns = not significant. ANOVA for (A) and Tukey’s multiple comparisons test for (C),(D), (E).

Given that SC grafts lacked follicular structures by day 16 post-transplantation (Fig. S1), we aimed to determine the time point at which germ cells disappeared within these grafts. Our findings indicated a reduction in the number of Ddx4-positive cells in SC-grafts as early as day two, with nearly complete disappearance by day eight (Fig. S2A and S2B). Consequently, we hypothesized that the subcutaneous location might influence the meiotic entry of germ cells in the transplanted gonads. To investigate this, we examined the expression of the Stra8 through immunofluorescence staining of grafts on the second and fourth days after transplantation, as the entry of germ cells into meiosis is crucially dependent on Stra8 expression [31, 32]. However, we observed no significant changes in the number of Stra8-positive cells in SC grafts on days 2 and 4 post-transplantation compared to KC grafts and *in vitro* cultured gonads (Fig. 2A). Thus, SC location appears to have minimal effects on the onset of meiosis in germ cells within the transplanted gonads.

Next, we investigated the impact of SC placement on the meiotic progression of germ cells in transplanted gonads. Co-immunostaining of meiocyte spreads with the synapsis proteins SYCP3 and SYCP1 revealed that meiotic prophase I progression was impaired in SC grafts 4 days post-transplantation (Fig. 2B). We observed that the proportion of SYCP3-positive cells in SC grafts was significantly lower than that in *in vitro* cultured gonads or KC-implanted grafts (Fig. 2C), which was consistent with the counting results of Ddx4-positive germ cells shown in Fig. S2B. Normal levels of pairing and synapsis were recorded in *in vitro* cultured gonads (97.3% ± 1.853%) and in KC grafts (94.82% ± 1.732%) (Fig. 2D). At pachytene, 20 fully synapsed chromosomes were clearly visible (Fig. 2B). However, synapsis was incomplete in SC transplants, as evidenced by fragmented SYCP1/SYCP3 staining at the pachytene stage, with an abnormal level of synapsis recorded at 48.72% ± 10.01% (Fig. 2B and 2D). Additionally, we staged the cells and discovered that the germ cells at 4 days were primarily in the zygotene and pachytene stages (Fig. 2E). The meiotic development of germ cells in both SC and KC grafts was found to be more similar; however, the meiotic progression in both graft types appeared to be faster compared to that of germ cells from cultured gonads (Fig. 2E).

### Meiocytes in SC fetal gonad suffer from double strand breaks

**Figure 3.**
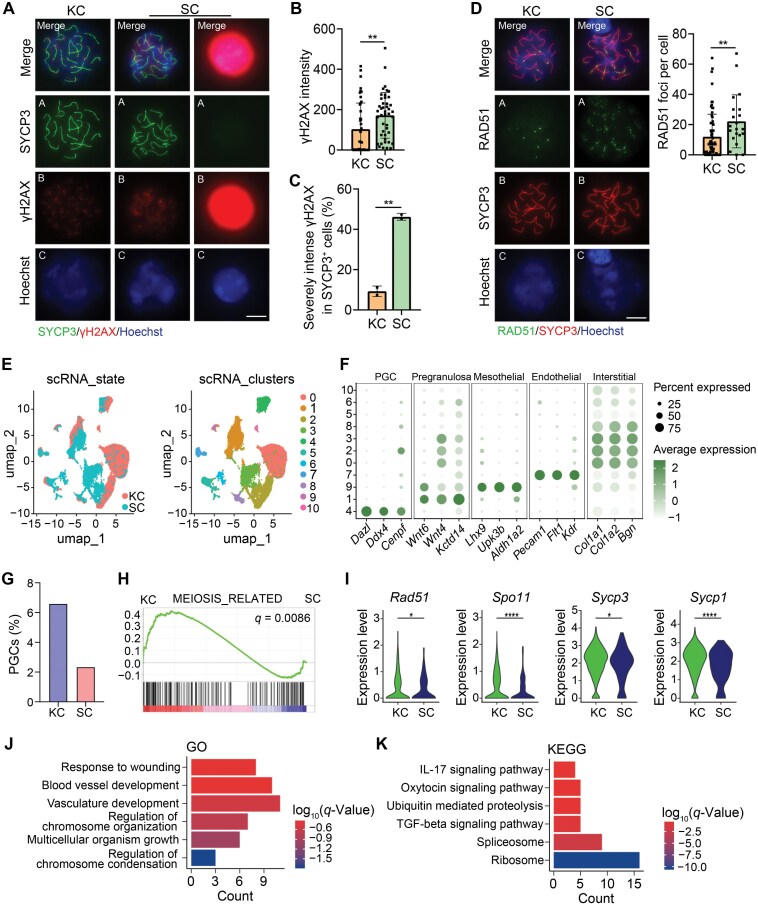
**Meiocytes in SC grafts with DSB repair defects during meiotic prophase I.**(A) Representative images of immunofluorescence staining of SYCP3 (green) and γH2AX (red) in pachytene stage meiocytes from KC transplants and SC transplants, respectively. Representative image of meiocytes with severe γH2AX signals in SC transplants. Scale bar: 10 μm. (B) Fluorescence intensity of γH2AX in KC transplants and SC transplants. Quantification of the fluorescence intensity in pachytene cells was performed using ImageJ under the same parameters. Data are presented as mean ± SD. (C) Percentage of meiocytes with severe γH2AX intensity in γH2AX-positive cells in KC and SC transplants. Mean ± SD. (D) Representative images of immunofluorescence staining of SYCP3 (red) and RAD51 (green) in pachytene stage meiocytes from KC transplants and SC transplants. Scale bar: 10 μm. Quantification of RAD51 foci number in pachytene stage meiocytes from KC transplants and SC transplants. (E) Two-dimensional (2D) visualization of clusters based on different samples (left) and transcriptional patterns (right) using UMAP. (F) Dot plot of the PGC, pregranulosa cell, mesothelial cell, endothelial cell and interstitial cell marker expression across all cell types. Each cell cluster is color-coded by identity. The dot size represents the percentage of cells expressing the indicated genes in each cluster, while dot color intensity reflects the average expression level of the indicated genes. (G) Percentage of PGC across different samples. (H) GSEA of meiosis-related pathways. (I) The VlnPlot showing differentially expressed genes associated with meiosis. (J) Top enriched GO terms of DEGs between KC and SC in PGC. (K) Top enriched KEGG terms of DEGs between KC and SC in PGC. Mean ± SD. *P < 0.05, **P < 0.01, ***P < 0.001, ns = not significant. Student’s *t* test for (B), (C), (D).

We assessed double-strand breaks (DSBs) levels by staining for phosphorylated histone H2AX (γH2AX) [33]. In germ cells from KC grafts, the γH2AX signal became restricted to the sex chromosomes in the pachytene and diplotene stages along with the gradual repair of the DSBs. In contrast, germ cells in the pachytene and diplotene stages from SC grafts displayed elevated γH2AX levels (Fig. 3A and 3B). Notably, over 45% of SYCP3-positive cells in SC grafts exhibited extremely intense γH2AX levels, significantly exceeding those observed in KC grafts (Fig. 3C). To determine whether DSB repair was compromised in SC grafts, we examined the localization of RAD51 [34]. In KC grafts, RAD51 protein was detected at numerous recombinase foci in late leptotene and zygotene germ cells, with a decrease in foci numbers during pachytene as DSB repair progressed. Conversely, in SC grafts, the number of RAD51 foci was moderately elevated in pachytene germ cells (Fig. 3D). These abnormalities contribute to the excessive DSBs observed in SC grafts.

To further investigate the underlying reasons for the severe meiotic defects in SC grafts, we performed 10× single-cell RNA sequencing on both KC grafts and SC grafts at day 4 post-transplantation. Following stringent data quality control and screening for Actin-GFP signals, we obtained a total of 13,659 cells from KC grafts and 7671 cells from SC grafts. Utilizing Seurat for UMAP dimensionality reduction analysis (Fig. 3E), we identified 11 distinct clusters. Based on differential gene expression among these clusters, they were classified into germ cells, pre-granulosa cells, mesothelial cells, endothelial cells, interstitial cells, and others (Fig. 3F). Statistical analysis revealed a significantly lower proportion of germ cells in SC grafts compared to KC grafts (Fig. 3G), consistent with our previous observation that the number of germ cells decreased significantly by day 4 in SC transplants (Fig. S2). Gene set enrichment analysis (GSEA) revealed downregulation of the meiosis-related pathway in SC transplants (Fig. 3H). Further examination of differentially expressed genes in renal cysts and SC transplants demonstrated significant downregulation of key meiotic proteins, including *Sycp3* and *Sycp1* (Fig. 3I), as well as defects in DSB-related proteins *Rad51* and *Spo11*, which align with our previous findings (Figs. 2B and 3D). To elucidate the underlying mechanism behind this profound impairment of meiosis, we conducted GO and KEGG analyses on enriched pathways. The GO analysis indicated abnormalities in processes such as vascular development and chromosome organization in SC transplants (Fig. 3J), while the KEGG analysis suggested defects in IL-17 signaling pathway, ribosome biogenesis, and other pathways (Fig. 3K) [35–37]. Thus, it is plausible that the compromised ability to undergo normal meiosis observed in SC transplants may be associated with impaired blood vessel formation and immune inflammatory responses.

### Temperature and oxygen concentration alter meiosis in SC fetal gonad grafts

**Figure 4.**
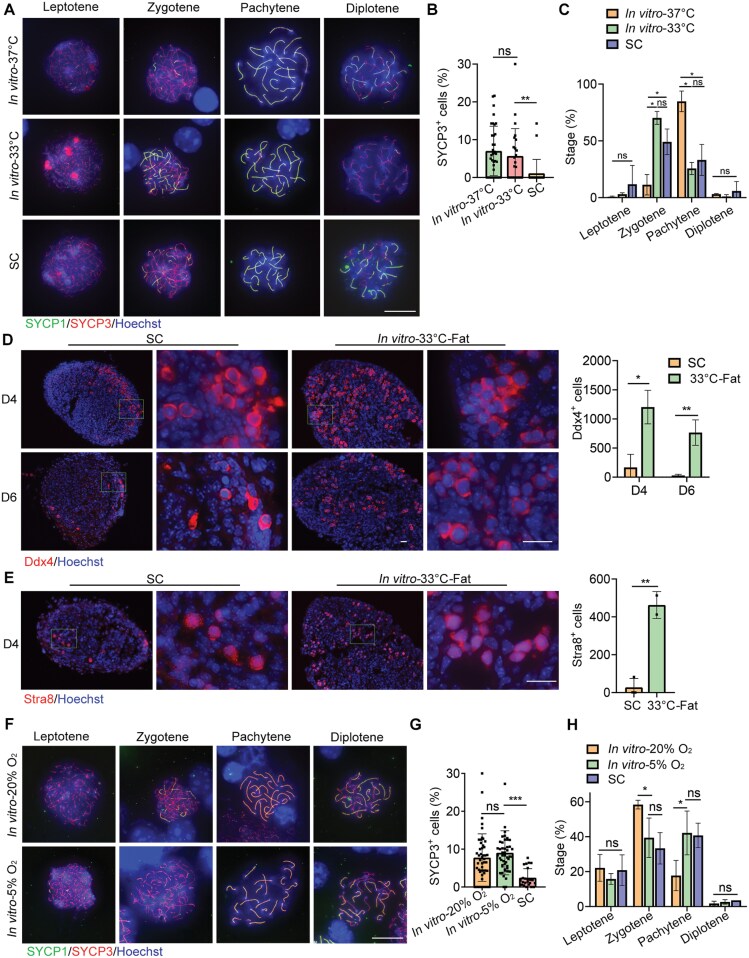
**The effect of temperature and oxygen concentration on meiotic prophase I in SC grafts.**(A) Meiocyte spreading and immunofluorescence staining of SYCP1 (green) and SYCP3 (red) in gonads cultured at 37°C, gonads cultured at 33°C and six-day SC transplants. Scale bar: 10 μm. (B) Percentage of SYCP3-positive cells in SC-transplanted gonads at 6 days, compared to gonads cultured at 37°C and 33°C. The proportion of SYCP3^+^ cells to total cells was calculated in each field of view. Mean ± SD. (C) Meiotic cells at various developmental stages in SC grafts and gonads cultured at 37°C and 33°C, respectively. Mean ± SD. (D) Immunofluorescence staining of Ddx4 in gonads co-cultured with fat at 33°C for 4 and 6 days. Scale bar: 20 µm. The number of Ddx4-positive cells was counted. Mean ± SD. (E) Immunofluorescence staining of Stra8 in gonads co-cultured with fat at 33°C for 4 days. Scale bar: 20 µm. The number of Stra8-positive cells was counted. Mean ± SD. (F) Meiocyte spreading and immunofluorescence staining of SYCP1 and SYCP3 in gonads cultured in 20% oxygen and 5% oxygen, respectively. Scale bar: 20 µm. (G) Percentage of SYCP3-positive cells in SC-transplanted gonads at 6 days and in gonads cultured in 20% oxygen and 5% oxygen. (H) Meiotic cells at various developmental stages in SC grafts and in gonads cultured in 20% oxygen and 5% oxygen, respectively. Mean ± SD. *P < 0.05, **P < 0.01, ***P < 0.001, ns = not significant. Tukey’s multiple comparisons test for (B), (C),(G), (H). Student’s *t* test for (D),(E).

To better understand the causes of anomalies and meiotic malformations in SC grafts, we investigated the potential impacts of temperature [38], oxygen content [38–41], and the surrounding composition beneath the skin [42]. SC temperature was monitored to approximately 33°C using an infrared thermometer. To assess the temperature dependence of meiotic progression in germ cells, we incubated the gonads *in vitro* at 33°C and 37°C for 6 days. Our observations revealed that the number of SYCP3-positive cells in the gonads cultured at 33°C did not differ from that in the gonads incubated at 37°C but was greater than that in the SC grafts (Fig. 4A and 4B). Additionally, the percentages of leptotene, zygotene, pachytene, and diplotene did not differ between the gonads incubated at 33°C and the SC grafts. However, in the gonads incubated at 37°C, the percentage of zygotene was lower, while the percentage of pachytene was higher (Fig. 4C). Therefore, SC temperature partially contributed to meiotic failure in the SC grafts.

Furthermore, gonads were co-cultured *in vitro* with SC white adipose tissue at 33°C. We found that the number of Ddx4-positive cells in the incubated gonads was considerably larger on days 4 and 6 compared to the SC grafts (Fig. 4D). Consistent with this finding, the number of Stra8-positive cells in the gonads co-cultured with adipose tissue at 33°C on day 4 was also greater than that in the SC grafts (Fig. 4E). Thus, there appear to be additional unknown factors contributing to germ cell apoptosis in SC transplanted gonads, beyond the influence of SC temperature.

Given that the SC environment is hypoxic [39–41], we then investigated the meiotic process of germ cells in the cultured gonads *in vitro* at 5% oxygen content on day 4. Compared to the cultured gonads *in vitro* at 20% oxygen content, the number of SYCP3-positive cells in the gonads cultured at 5% oxygen content did not differ from the gonads incubated at 20% oxygen content, but was greater than that in the SC grafts (Fig. 4F and 4G). However, the percentages of leptotene, zygotene, pachytene, and diplotene did not differ between the gonads incubated at 5% oxygen and the SC grafts. In contrast, in the gonads incubated at 20% oxygen, the percentage of zygotene was higher, while the percentage of pachytene was lower (Fig. 4H). Therefore, a hypoxic environment might affect germ cell meiosis in SC grafts.

### Short-term *in vitro* culture of fetal gonads enhances folliculogenesis of fetal gonads following SC transplantation

To mitigate meiotic defects associated with SC transplantation for gonad development and follicle formation, we hypothesized that short-term culture would promote folliculogenesis of fetal gonads following SC transplantation. Given the robust progression of meiotic prophase observed from days 5 to 9 in *in vitro* incubated rOvaries, and the substantial induction of primary oocytes from PGCs under the employed culture conditions [25], we dissociated E12.5 female gonads from albino ICR mice and reaggregated germ cells with somatic cells in low-binding U-bottom 96-well plates for 2 days to form rOvaries (D0) and cultured them in collagen I coated transwell membranes (Fig. S3A). After 7 days of culture, follicle-like structures successfully assembled (Fig. S3B). The number of follicle-like structures increased and gradually developed over the following days, with visible secondary follicle structure observed after 20 days of culture (Fig. S3B and S3C). In the rOvaries cultured for 20 days, primary and secondary follicles predominated, with only a few primordial and mature follicles present (Fig. S3B). Additionally, compared to the GK15 reconstituted medium, the MF10 medium supplemented with Rock inhibitor and vitamin C was more effective in promoting the reconstitution of gonad cells and subsequent follicle development (Fig. S3D–F).

The rOvaries were subcutaneously transplanted after 6 days of *in vitro* culture. The presence of follicles and the expression of germ cell and granulosa cell markers were evaluated 10 and 25 days post-SC transplantation. Oocytes with Actin-GFP fluorescence were observed in the grafts at both 10 and 25 days after transplantation (Fig. S4A). At day 10, Foxl2-positive cells surrounded Ddx4-positive cells (Fig. S4B). By day 25, secondary follicle structures, composed of Ddx4-positive oocytes and multilayered Foxl2-positive granulosa cells, had formed (Fig. S4B). Histology analysis using H&E staining revealed follicle-like structures in the 10-day and 25-day grafts, containing primary, secondary and antral follicles (Fig. S4C). The number of follicles in the 10-day grafts did not differ from that in the 25-day grafts (Fig. S4D). However, the percentages of both primordial and primary follicles in the 10-day grafts was significantly higher than those in the 25-day grafts. In contrast, secondary and antral/mature follicles predominated in the 25-day grafts (Fig. S4E).

**Figure 5.**
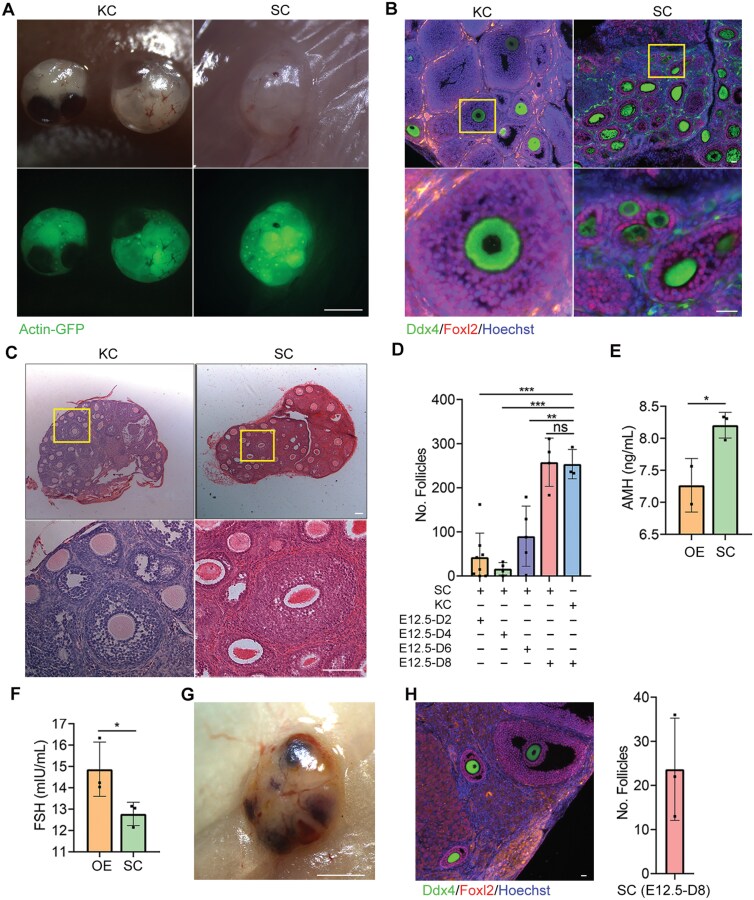
**Folliculogenesis of short-term cultured fetal gonads following SC transplantation.**(A) Morphology of 25-day grafts at both the KC and SC sites after the transplantation of an 8-day *in vitro* cultured gonad. Scale bar: 1 mm. (B) Immunofluorescence staining of Ddx4 and Foxl2 in 25-day grafts at both KC and SC sites following the transplantation of an 8-day *in vitro* cultured gonad. Scale bar: 20 µm. (C) H&E staining of a 25-day graft at both the KC and SC sites after transplantation of an 8-day *in vitro* cultured gonad. Scale bar: 100 µm. (D) Follicle count in 25-day grafts at the SC site after the transplantation of 2-, 4-, 6- and 8-day *in vitro* cultured E12.5 gonad. *n* ≥ 3. Mean ± SD. (E) Serum AMH levels in mice with SC grafts compared to mice with bilateral OE. *n* = 3. Mean ± SD. (F) Serum FSH levels in mice with subcutaneous grafts compared to mice with bilateral OE. *n* = 3. Mean ± SD. (G) Morphology of an 8-week graft at the SC site following the transplantation of an 8-day *in vitro* cultured gonad. Scale bar: 1 mm. (H) Immunofluorescence staining of Ddx4 and Foxl2 in 8-week grafts at the SC site after the transplantation of an 8-day *in vitro* cultured gonad. Scale bar: 20 µm. The number of follicles in 8-week gonads at the SC site transplanted under the skin for 8 weeks. *n* ≥ 3. Mean ± SD. *P < 0.05, **P < 0.01, ***P < 0.001, ns = not significant. Tukey’s multiple comparisons test for (D). Student’s *t* test for (E), (F).

Next, we dissociated E12.5 female gonads and cultured them *in vitro* for 2, 4, 6, and 8 days before transplanting them into allogenic ovariectomized mice via SC transplantation. Grafts cultured *in vitro* for 8 days and subsequently SC-transplanted 25 days later exhibited numerous oocytes with Actin-GFP fluorescence (Fig. 5A). Immunofluorescence staining analysis revealed that Foxl2-positive granulosa cells surrounded Ddx4-positive oocytes to generate follicular structures (Fig. 5B). Histology examination of 25-day graft sections by H&E staining indicated that the number of follicles in the grafts cultured *in vitro* for 8 days was comparable to that of the kidney capsule transplantation (Fig. 5C). In contrast, grafts cultured *in vitro* for 2, 4, and 6 days contained significantly fewer follicles (Fig. 5D). Given that the follicle development of the gonads cultured *in vitro* for 2, 4, and 6 days did not achieve the same outcomes as KC transplantation (Fig. 5D), we sought to determine whether at least 8 days of *in vitro* culture were necessary for meiotic prophase I to be arrested until oocyte meiosis resumed. We found no differences in the expression of Ddx4 and Foxl2 markers in gonads cultured for 6 and 8 days (Fig. S5A). However, SYCP3 was detectable at 6 days of culture but became undetectable by 8 days (Fig. S5B). Thus, gonads cultured *in vitro* for 8 days likely reached the diplotene stage or completed the meiotic prophase I process. This strategy may enhance folliculogenesis of gonads following subcutaneous transplantation. Furthermore, we performed an ELISA to examine whether SC grafts could potentially restore endocrine functions in mice that underwent bilateral ovariectomy (OE). In mice with SC grafts, serum follicle-stimulating hormone (FSH) levels were lower, while anti-Mullerian hormone (AMH) levels were higher compared to mice with bilateral OE (Fig. 5E and 5F), indicating that SC grafts possess the ability to generate and release hormones. A few follicles remained present in each gonad graft 8 weeks after SC transplantation (Fig. 5G and 5H). We also investigated the presence of functional oocytes in both KC grafts and SC grafts. On day 25 post-transplantation, the grafts were punctured with a needle to retrieve GV (germinal vesicle) stage oocytes for subsequent *in vitro* maturation and fertilization experiments. The results demonstrated successful maturation and attainment of the blastocyst stage after fertilization for both KC grafts and SC grafts (Fig. S6A), although the proportion of oocytes reaching the blastocyst stage was lower in SC grafts (Fig. S6B). However, upon transferring 2-cell embryos from each group into pseudo-pregnant mice (Fig. S6C), only KC graft-derived embryos exhibited successful development into viable mice (Fig. S6D).

## Discussion

**Figure 6.**
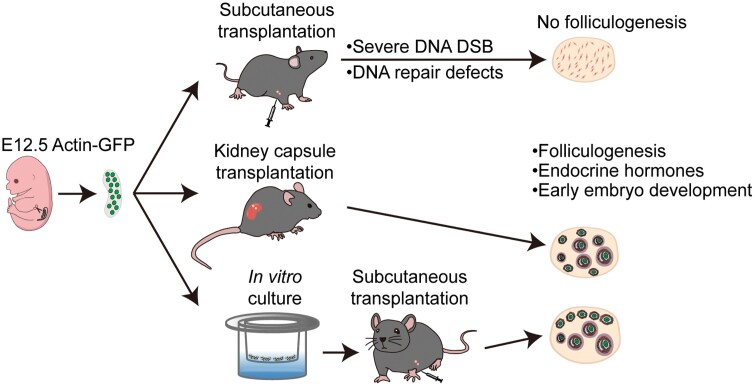
**Follicular development of fetal gonads under the skin of adult mice.**Follicular development in fetal gonads under adult mouse skin is hindered by meiotic defects due to unsuitable temperature and oxygen levels. However, short-term *in vitro* culture of E12.5 female gonads in optimal conditions allows them to complete meiosis, enabling functional egg formation after subcutaneous transplantation.

In this study, we investigated the potential for follicular development in fetal gonads implanted under the skin of adult mice (Fig. 6). Our findings indicate that KC-implanted gonads successfully undergo folliculogenesis, whereas SC transplanted E12.5 gonads fail to develop follicular structures. We demonstrate that the SC-transplanted gonads experience meiotic defects. Furthermore, we identify several contributing factors to the anomalies and meiotic malformation observed in SC grafts, including SC temperature, oxygen concentration, and the surrounding tissue composition. We observe robust progression of meiotic prophase occurring from days 6 to 8 *in vitro*-incubated E12.5 female gonads. Additionally, we demonstrate that subcutaneous transplantation of short-term *in vitro* cultured E12.5 female gonads promotes the folliculogenesis and restores endocrine functioning.

Several studies have reported that the cryopreservation of ovarian tissue followed by reimplantation may restore ovarian endocrine function and prevent menopause-related conditions [6, 7]. The transplantation sites, including intraperitoneal, ovarian bursa, KC, and SC, have been reported to yield similar outcomes concerning follicular density, growth and morphology, and endocrine function [43]. Among these procedures, SC-transplantation is less invasive and can potentially be performed under local anesthesia, with the primary aim of restoring sex steroid secretion post-menopause. Notably, ovarian biopsies should ideally be taken when follicular density is high (between the ages of 20 and 25 years), as this facilitates long-term restoration of ovarian function [44, 45]. In mammals, oogonia differentiate into oocytes, entering the first prophase of meiosis in response to signals, particularly from pregranulosa cells. Additionally, female mouse germ cells from E12.5 gonadal ridges exhibit greater meiotic potential compared to E11.5 germ cells under *in vitro* conditions [46]. These findings prompted us to evaluate the outcomes of folliculogenesis following the transplantation of E12.5 gonads into both KC and SC sites. Our study demonstrated that KC-implanted gonads successfully undergo folliculogenesis, corroborating previous research that indicates meiosis induction and folliculogenesis occur through the aggregation of PGCs or PGCLCs with E12.5 somatic cells, followed by kidney capsule transplantation [14, 17, 19, 26, 27]. Moreover, the ratios of secondary follicles increased in the *in vitro*-cultured and KC-implanted groups, attributed to the premature activation of primordial follicles [14, 20]. Furthermore, our previous work demonstrated that mTOR inhibition by INK128 extends the follicular and endocrine functions of the KC-transplanted rOvaries in recipient mice [20]. Additionally, we observed that meiotic progression in both SC-transplanted and KC-transplanted mouse fetal gonads was more rapid 4 days post-transplantation than in germ cells derived from cultured gonads. Previous studies have indicated that the activation of primordial follicles from oocytes is a complex process influenced by various regulatory factors, hormones, and cytokines [47, 48]. The absence of essential cytokines and hormones in the culture media often contributes to the delayed activation of primordial follicles. Notably, our data indicated that SC transplanted E12.5 gonads fail to develop follicular structures due to meiotic defects. These defects may be attributed to the relatively poor vascularization and a stressful environment sensitive to changes in external temperature and pressure.

The E12.5 female gonads cultured *in vitro* for 8 days reached the diplotene stage or completed the meiotic prophase I process. Oocytes, arrested at the diplotene stage of meiotic prophase I, are encased by a single layer of granulosa cells to form primordial follicles, which serve as the source for oogenesis. Moreover, this strategy promotes folliculogenesis of gonads following SC transplantation. We observed that primordial follicles are resistant to stressful environments, whereas growing follicles appear to be more sensitive to both cryoinjury and hypoxic environments occurring shortly after transplantation. Accordingly, unlike the mean numbers of follicles from grafted ovarian tissue in both KC and SC groups [49], the number of follicles in the SC grafts that were cultured *in vitro* for 8 days was comparable to that of the KC transplantation. Recently, Saitou et al. demonstrated an appropriate *in vitro* maturation of fetal ovaries mainly with oogonia into those bearing primordial follicles both in humans and in monkeys. However, the proportion of oogonia that differentiated into oocytes in primordial follicle-like structures appeared to be lower, and the time required for such differentiation was longer under *in vitro* conditions [24]. Encouragingly, methods have been developed to generate both human primordial germ cell-like cells (hPGCLCs) and functional human ovarian granulosa-like cells from human induced pluripotent stem cells have been developed [23, 50–52]. Moreover, human ovarian granulosa-like cells aggregated with hPGCLCs form ovarioids and support hPGCLC development from the promigratory to the gonadal stage [23]. Thus, our findings suggested that SC transplantation of fetal gonad like structures and subsequent development of ovarioids may provide an alternative prospective therapy strategy for intervening menopause and associated chronic diseases, thus improving healthspan.

## Research limitations

Our study demonstrated that SC grafts exhibit meiotic abnormalities and significant double-strand breaks during meiosis. This is partly due to the low oxygen environment and nutritional starvation. Furthermore, the eggs retrieved from SC transplant were fertilized *in vitro*, yet no pups were obtained after embryo transplantation, indicating that the SC transplantation cannot fully replace kidney transplantation. Further research is necessary to develop a more effective SC graft model.

## Methods

### Experimental animals

C57BL/6N-Tg (CAG-EGFP) C14-Y01-FM131Osb (beta-Actin-GFP) mice were purchased from the Model Animal Research Center of Nanjing University. NOD-SCID mice, C57BL/6NCrSlc and albino ICR mice were sourced from Beijing Vital River Laboratory Animal Technology Co., Ltd. Mice aged 4–8 weeks were healthy and housed in individually ventilated cages under a standard 12-h light/12-h dark cycle in the sterile Animal Facility at the College of Life Sciences. Eight-week-old female mice were used for the rOvaries or cultured gonads auto transplantation model.

### rOvaries and gonad culture

rOvaries were reaggregated and cultured as previously described [25, 53]. Briefly, PGCs from C57BL/6-GFP or ICR fetal ovaries were aggregated with E12.5 female gonadal somatic cells from the same gonads in a low-binding U-bottom 96-well plate (Sigma-Corning) for 2 days of culture in GMEM medium containing 15% KnockOut Serum Replacement (KSR, Invitrogen), 1 mM glutamine, 50 units/mL penicillin and 50 μg/mL streptomycin (2A, Invitrogen), 1 mM nonessential amino acids, 55 μM 2-mercaptoethanol (Invitrogen) and 3 μM retinoic acid (GK15 medium) or M199 (Sigma) containing 10% FBS (ES quality, HyClone), 2 mM L-glutamine, 1% nonessential amino acid stock, 50 units/mL penicillin and 50 μg/mL streptomycin, 50 μg/mL ascorbic acid (vitamin C, Vc, Sigma), and 10 μM Rocki (MF10 medium). Each aggregate contained 55,000 gonadal cells [16].

Two days later, rOvaries or E12.5 intact gonads were transferred by a glass capillary to collagen I-coated transwell membranes (Millipore) and cultured in αMEM medium (Thermo Scientific) containing 2% FBS, 50 μg/mL ascorbic acid, 2 mM glutamine, 50 units/mL penicillin, 50 μg/mL streptomycin, and 55 μM 2-mercaptoethanol (αMEM-based IVDi). After 4 days, the culture medium was changed to StemPro-34 SFM (Gibco) medium supplemented with 10% FBS, 50 μg/mL ascorbic acid, 1 × glutamine, 50 units/mL penicillin, 50 μg/mL streptomycin, and 55 μM 2-mercaptoethanol (StemPro-34-based IVDi medium). From days 7 to 11 of culture, 500 nM ICI 182,780 was added to the medium, and the medium was changed every 2 days.

### Transplantation

KC transplantation was performed according to previously established methods [17]. Briefly, one aggregate was swiftly placed into the “pocket,” which was made between the KC and kidney tissue of a bilaterally ovariectomized recipient mouse. The entire transplantation procedure was completed in 5 min for each mouse. Meiosis and folliculogenesis were observed in the reconstituted ovaries 25 days after the transplantation of the aggregates.

SC transplantation was performed by aspirating two to four gonads or rOvaries in 200 microliters of MF10 medium with a syringe. The mixture was then injected into the SC side of the back of anesthetized mice. Subsequently, the recipient mouse ovaries were surgically removed. The grafts were removed at the proper time.

### Tissue collection and H&E staining

Tissues were carefully collected and fixed overnight in 3.7% paraformaldehyde (PFA) at 4°C. The tissues were sequentially dehydrated with varying concentrations of ethanol for 1 h each and treated twice with xylene for 30 min each. Then, the samples were immersed in wax three times for 1 h each. The tissue was then embedded using an automatic paraffin tissue embedding machine and stored at 4°C.

For follicle counting, the tissue was sequentially sectioned, and every fifth slide was collected on glass microscope slides for H&E staining. Sections were cut to a thickness 5 μm, dried at 65°C, and stored at room temperature. The tissue sections were deparaffinized three times in xylene for 10 min each, rehydrated with different concentrations of ethanol, washed in distilled water, stained with hematoxylin and eosin Y for the appropriate duration, and subsequently washed with distilled water, varying concentrations of ethanol and xylene, and sealed with neutral resin.

### Fluorescence microscopy

Tissue sections were deparaffinized in xylene three times for 10 min each, rehydrated with various concentrations of ethanol, and washed in PBS. The sections were incubated in citrate buffer for 3 min at 120°C for antigen retrieval, permeabilized in PBST (0.1% Triton X-100 dissolved in PBS) for 10 min and blocked with 3% BSA for 2 h at room temperature. Subsequently, they were incubated with primary antibodies overnight at 4°C. The primary antibodies were used for immunocytochemistry included anti-Ddx4/Vasa/MVH, Foxl2, Stra8, and SYCP3. After three washes with PBS, the sections were incubated with secondary antibodies for 2 h at room temperature. The secondary antibodies used for immunocytochemistry were Alexa Fluor® 594 Donkey Anti-Rabbit IgG (H + L), Alexa Fluor® 488 Donkey Anti-Mouse IgG (H + L), Alexa Fluor® 594 Donkey Anti-Goat IgG (H + L), and Alexa Fluor® 488 Donkey Anti-Rabbit IgG (H + L). Finally, the sections were incubated with 1 μg/mL Hoechst 33342 for nuclear staining and sealed with Vectashield.

### Fluorescence microscopy of meiocyte spreads

Gonads or reconstituted ovaries were digested into single cells using 0.25% trypsin-EDTA (TE, Invitrogen) at 37°C for 7 min. The cells were then resuspended in 100 mM sucrose and spread onto glass slides coated with a layer of fixative. After three hours, the slides were washed with 0.04% Photo-Flow (Kodak) and dried at room temperature. The slides were permeabilized in 0.1% Triton X-100 for 10 min, blocked with ADB (3% BSA and 2% goat serum dissolved in PBST) for 2 h at room temperature, and incubated overnight at 4°C with anti-SYCP1, anti-SYCP3, anti-RAD51 or anti-γH2AX antibodies. The slides were then washed three times and incubated with Alexa Fluor® 594 Donkey Anti-Rabbit IgG (H + L) and Alexa Fluor® 488 Donkey Anti-Mouse IgG (H + L) secondary antibodies. Sections were incubated with 1 μg/mL Hoechst 33342 for nuclear staining and mounted using Vectashield mounting medium. All fluorescence samples were analyzed using an Axio-Imager Z2 fluorescence microscope.

### Single-cell RNA sequencing data processing

The DNBelab C4/DNBelab TaiM4 Series Single-Cell Library Prep Set (MGI) was used for sequencing. DNBs were loaded into the patterned nanoarrays and sequenced on the DNBSEQ-T7 sequencer with pair-end sequencing. The sequencing reads contained 30-bp read 1 (including the 10-bp cell barcode 1, 10-bp cell barcode 2 and 10-bp unique molecular identifiers [UMI]), 100-bp read 2 for gene sequences and 10-bp barcodes read for sample index. The sequencing data were processed using an open-source pipeline (github.com/MGI-tech-bioinformatics/DNBelab_C_Series_scRNA-analysis-software). The DNBC4tools software was obtained from github (github.com/MGI-tech-bioinformatics/DNBelab_C_Series_HT_scRNA-analysis-software). Alignment, filtering, barcode counting, and UMI counting were performed with DNBC4tools count module to generate the feature-barcode matrix and identify clusters.

### Single-cell RNA sequencing data analysis

Import the raw count matrix into R for further processing. Run R scripts using R studio for hierarchical clustering and PCA. The count matrix was initially normalized by the library size and logarithmically transformed by Seurat V5. The transcriptomes with fewer than 1000 expressed genes were excluded, while cells with mitochondrial genes occupying > 25% reads were defined as low-quality cells and filtered out. Use Seurat’s “Merge” function to integrate different samples according to the instructions. UMAP is used for visualization and clustering. The “FindAllMarkers” function was used to identify specific cell type markers under different conditions. The “FindMarkers” function was used to identify differential genes in germ cells between different samples, and the “VlnPlot” function was used to map the expression of target genes.

### *In vitro* maturation and *in vitro* fertilization

The grafts were dissected from the KC or skin of C57BL/6J female mice, and fully-grown GV oocytes were collected under a microscope by pricking the follicles with an insulin syringe in *in vitro* maturation (IVM) medium. Oocytes were matured *in vitro* by culturing in IVM medium for 17–18 h at 37°C [54]. The IVM medium contains a-MEM (Invitrogen) supplemented with 5% FBS, 0.24 mM sodium pyruvate, 1 IU/mL PMSG and 1.5 IU/mL human chorionic gonadotropin (hCG, Sigma). Oocytes at MII stage, identified by extrusion of the first polar body, were subjected to *in vitro* fertilization (IVF). For IVF, spermatozoa were collected from the C57BL/6J males, capacitated by incubation for 2 h in HTF, and then incubated with the matured oocytes for 6 h. The zygotes were transferred into KSOM medium. Embryos that reached the 2-cell stage after 24 h of culture were either transferred into the oviducts of E0.5 pseudo-pregnant mice or cultured in KSOM medium until the blastocyst stage, and pups were collected on E12.5.

### Research ethics

All animal experiments were complied with all relevant ethical regulations for animal testing and research and were in accordance with protocols approved by the institutional Animal Care and Use Committee of Nankai University (License number 20140006).

### Statistical analysis

Statistics were analyzed using GraphPad Prism software. Data were assessed with a two-tailed unpaired Student’s *t* test for comparison between two groups, or ANOVA for comparisons involving more than two groups, and are presented as mean ± SD. *P* values less than 0.05 were considered significant (*P < 0.05, **P < 0.01 or ***P < 0.001). “*n*” refers to the number of biological replicates, all experiments were repeated at least three times unless otherwise specified.

## Supplementary data

Supplementary data is available at *Life Medicine* online.

The key resources table summarizes the critical resources and materials used in this work (see supplementary material).

lnaf007_suppl_Supplementary_Figures

lnaf007_suppl_Supplementary_Material

## Data Availability

The accession number for single-cell RNA sequencing data reported in this paper is NCBI GSE282158.
