# Supplementary material for: Follicular development of fetal gonads under the skin of adult mice
Source: Life Med. 2025 Feb 24;4(3):lnaf007. doi: 10.1093/lifemedi/lnaf007 (PMC12084806; doi:10.1093/lifemedi/lnaf007)
Supplement: lnaf007_suppl_Supplementary_Figures [file lnaf007_suppl_supplementary_figures.pdf]

Follicular development of fetal gonads under the skin of adult mice

Jiyu Chen[1,2,#], Chang Liu[1,2,#], Yongqin Yu[1,2], Xiaoying Ye[1,2], Lin Liu[1,2,3,*], Zhengmao Zhu[1,2,3,*]

[1]Department of Genetics and Cell Biology, College of Life Science, Nankai University, Tianjin 300071, China
[2]State Key Laboratory of Medicinal Chemical Biology, Nankai University, Tianjin 300350, China
[3]Haihe Laboratory of Cell Ecosystem, Chinese Academy of Medical Sciences & Peking Union Medical College, Tianjin 300020, China

[#]These authors contributed equally to this work.
[*]Correspondence: liulin@nankai.edu.cn (L.L.), zhuzhengmao@nankai.edu.cn (Z.Z.)

Figure S1. Developmental defects of E12.5 gonads following subcutaneous transplantation.

(A) Morphology of SC grafts after transplantation for 16 days and 8 weeks. (B) Sixteen-day and 8-week subcutaneous grafts were embedded in paraffin, and 5-μm-thick sections were prepared and stained with H&E. Scale bar: 100 μm.

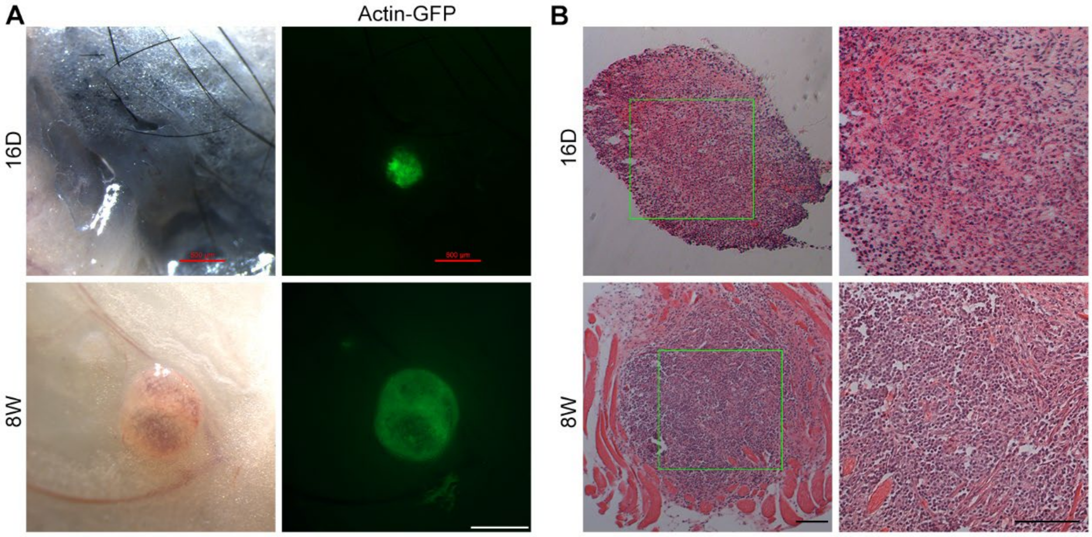

Figure S2. Decrease in germ cell number during development in subcutaneous grafts.

(A) Immunofluorescence staining of Ddx4 in gonads cultured *in vitro*, KC and SC grafts at 2, 4, 6 and 8 days. Scale bar: 20 μm. (B) Number of Ddx4-positive cells in gonads cultured *in vitro*, KC and SC grafts at 2, 2, 6 and 8 days. Ddx4-positive cells were quantified by aggregating the counts from every fifth section throughout the entire tissue. $n \geq 3$. Mean ± SD. *$P < 0.05$, **$P < 0.01$, ***$P < 0.001$, ns = not significant. Tukey's multiple comparisons test for (B).

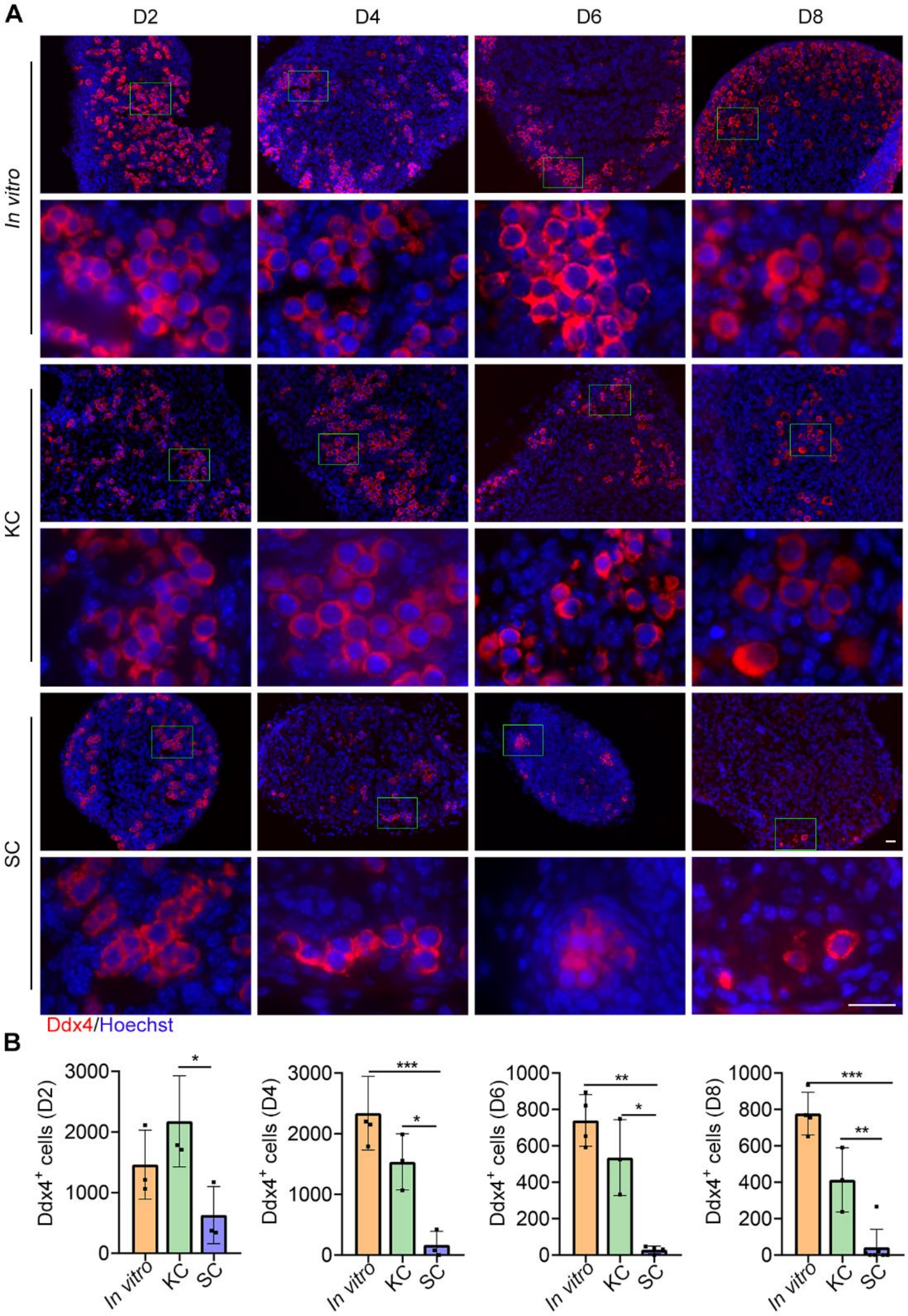

Figure S3. Follicle development of E12.5 gonads *in vitro*.

(A) Morphology of rOvaries reunited for one day and two days *in vitro* in various medium within a U-bottom 96-well plate. The culture media included GK15, GMEM plus 15% KSR. MF10 + Rocki + VC (MF10++), M199 plus 10% FBS, vitamin C and the Rock inhibitor ICI 182,780. Scale bar: 100 μm. (B) Morphology of rOvaries cultured in transwell during development *in vitro*. Scale bar: 100 μm. (C) Diameter changes of rOvaries during *in vitro* development. $n \geq 3$. (D) Twenty-day *in vitro* cultured rOvaries were embedded in paraffin, and sections were prepared and stained with H&E. Scale bar: 100 μm. (E) Number of follicles in 20-day cultured rOvaries within the GK15 or MF10 + Rocki + VC culture system. (F) Percentage of follicles at various developmental stages in 20-day *in vitro* cultured rOvaries. $n \geq 3$. Mean ± SD. $^{*}P < 0.05$, $^{**}P < 0.01$, $^{***}P < 0.001$, ns = not significant. Student's *t* test for (E), (F).

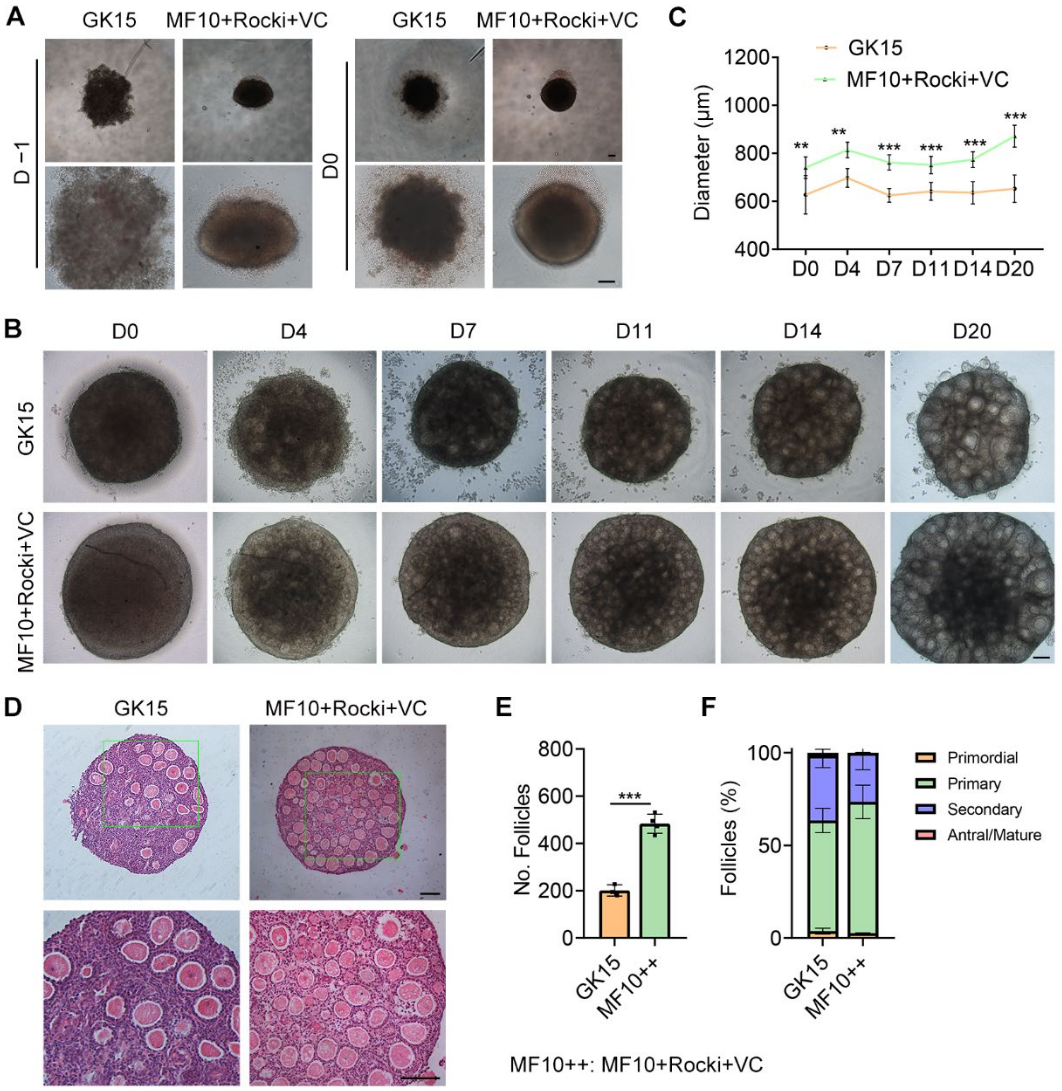

Figure S4. Folliculogenesis following subcutaneous transplantation of rOvary.

(A) Morphology of 10-day and 25-day grafts at the subcutaneous site after transplanting a 6-day *in vitro* cultured rOvary (D6–D10, D6–D25). Scale bar: 500 μm. (B) Immunofluorescence staining of Ddx4 and Foxl2 in D6–D10 and D6–D25 subcutaneous transplants. Scale bar: 20 μm. (C) H&E staining of D6–D10 and D6–D25 subcutaneous transplant sections. Scale bar: 100 μm. (D) Number of follicles of D6–D10 and D6–D25 subcutaneous transplants. $n \geq 3$. Mean ± SD. (E) Percentage of follicles at different developmental stages in D6–D10 and D6–D25 subcutaneous transplants. $n \geq 3$. Mean ± SD. $^{*}P < 0.05$, $^{**}P < 0.01$, $^{***}P < 0.001$, ns = not significant. Student's t test for (D), (E).

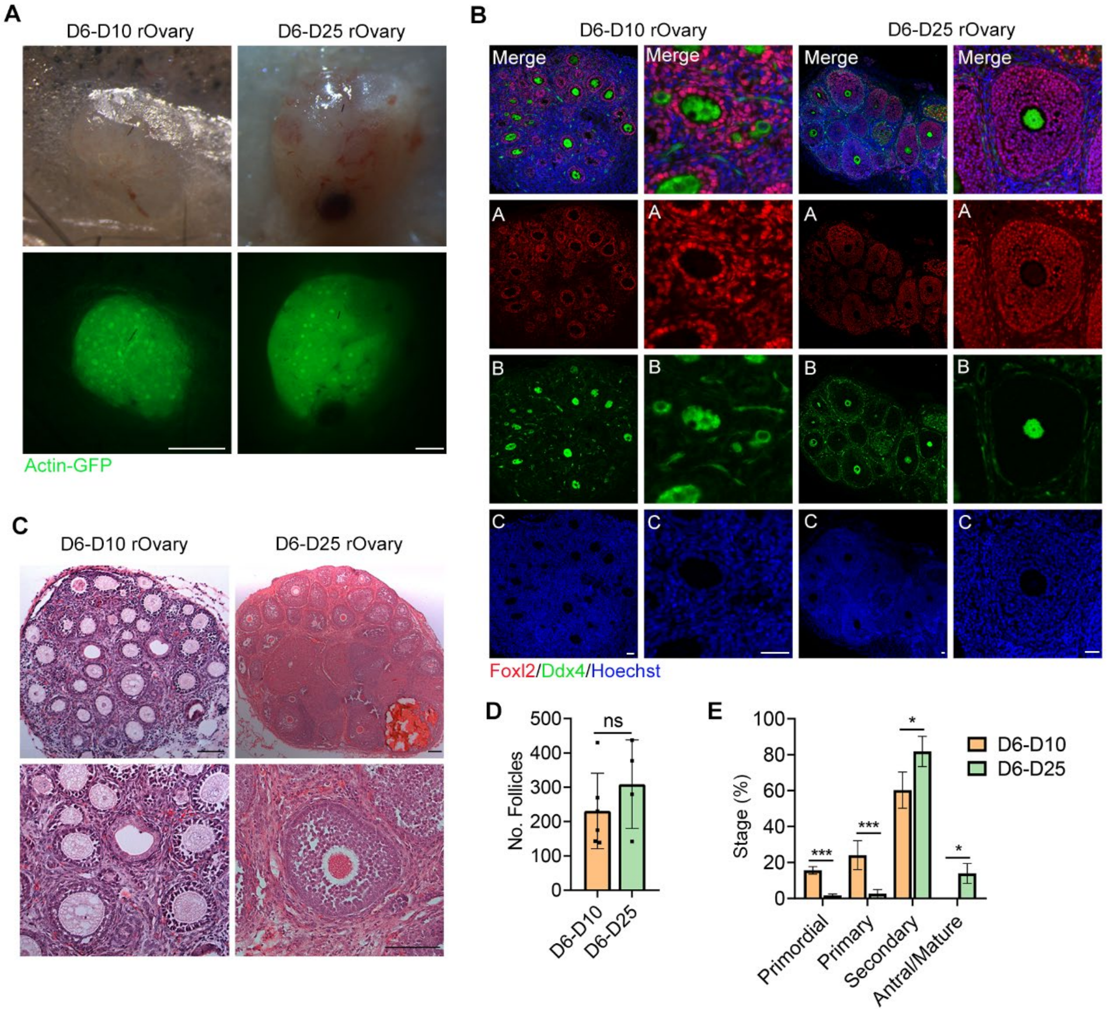

Figure S5. Completion of meiosis in 8-day *in vitro* cultured gonads. (A) Immunofluorescence staining of Ddx4 and Foxl2 in 6-day and 8-day cultured gonads. Scale bar: 20 μm. (B) Immunofluorescence staining of SYCP3 in 6-day and 8-day cultured gonads. Scale bar: 20 μm.

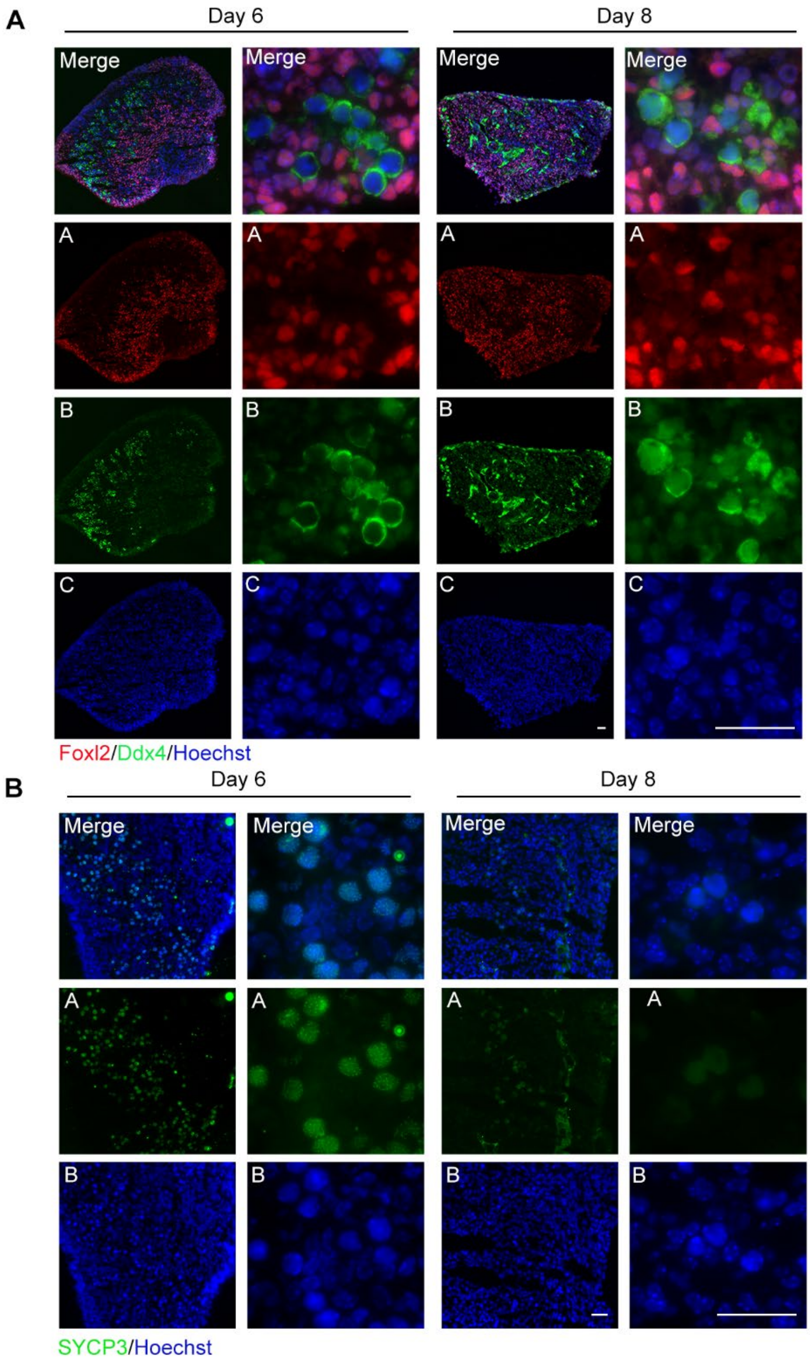

Figure S6. Follicles derived from the SC-transplanted fetal gonad fail to produce live offspring.

(A) GV oocytes isolated from control ovary, KC grafts, and SC grafts, along with embryo development following IVM and IVF. Scale bar: 100 μm. (B) Summary of GV oocytes development to the blastocyst stages. (C) Germline competency assessment of Control-ovary-derived, KC-grafts-derived, and SC-grafts-derived mice by injection 2-cell embryos into surrogate mothers. Pseudo-pregnant ICR mice served as surrogate mothers. (D) Summary of Control-ovary-derived, KC-grafts-derived, and SC-grafts-derived pups following the injection of the 2-cell embryos into surrogate mothers.

A C

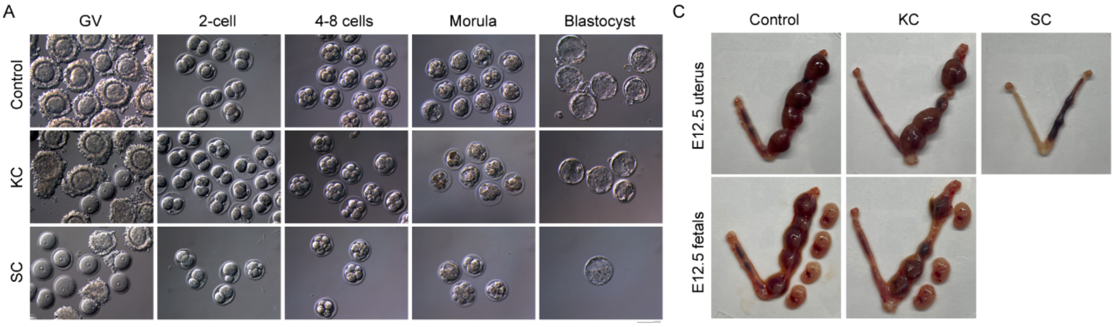

B

		No.GV Oocytes	No.2-cell(%)	No.4-8 cell(%)	No.Morula(%)	No.Blastocyst(%)
Control	1	44	16(36.36%)	15(34.09%)	13(29.55%)	10(22.73%)
	2	56	21(37.50%)	19(33.93%)	18(32.14%)	15(26.79%)
	3	40	22(55.00%)	22(55.00%)	19(47.5%)	12(30.00%)
KC	1	59	23(38.98%)	18(30.51%)	16(27.12%)	7(11.86%)
	2	50	12(24.00%)	8(16.00%)	7(14.00%)	3(6.00%)
	3	68	27(39.71%)	22(32.35%)	17(25.71%)	7(10.26%)
SC	1	54	8(14.81%)	5(9.26%)	4(7.41%)	0(0.00%)
	2	52	8(15.38%)	6(11.54%)	4(7.69%)	1(1.92%)
	3	60	6(10.00%)	3(5.00%)	3(5.00%)	1(1.67%)

D

		No.2-cell	No.Pups(%)
Control	1	7	4(57.14%)
	2	6	3(50.00%)
	3	7	3(42.86%)
KC	1	12	3(25.00%)
	2	11	1(9.09%)
	3	13	1(7.69%)
SC	1	6	0(0.00%)
	2	5	0(0.00%)
	3	7	0(0.00%)
