# Supplementary material for: Follicular development of fetal gonads under the skin of adult mice
Source: Life Med. 2025 Feb 24;4(3):lnaf007. doi: 10.1093/lifemedi/lnaf007 (PMC12084806; doi:10.1093/lifemedi/lnaf007)
Supplement: lnaf007_suppl_Supplementary_Material [file lnaf007_suppl_supplementary_material.docx]

Follicular development of fetal gonads under the skin of adult mice

Jiyu Chen^1,2,#^, Chang Liu^1,2,#^, Yongqin Yu^1,2^, Xiaoying Ye^1,2^, Lin Liu^1,2,3,^**^*^**, Zhengmao Zhu^1,2,3,^**^*^**

^1^Department of Genetics and Cell Biology, College of Life Science, Nankai University, Tianjin 300071, China

^2^State Key Laboratory of Medicinal Chemical Biology, Nankai University, Tianjin 300350, China

^3^Haihe Laboratory of Cell Ecosystem, Chinese Academy of Medical Sciences & Peking Union Medical College, Tianjin 300020, China

^#^These authors contributed equally to this work.

^∗^Correspondence: [liulin@nankai.edu.cn](mailto:liulin@nankai.edu.cn) (L.L.), [zhuzhengmao@nankai.edu.cn](mailto:zhuzhengmao@nankai.edu.cn) (Z.Z.)

**Key resources table**

| REAGENT or RESOURCE | SOURCE | IDENTIFIER |
| --- | --- | --- |
| Antibodies |  |  |
| Ddx4 | Abcam | Cat#: ab13840 |
| Foxl2 | Abcam | Cat#: ab5096 |
| Stra8 | Abcam | Cat#: ab49602 |
| SYCP1 | Abcam | Cat#: ab15090 |
| SYCP3 | Abcam | Cat#: ab97672 |
| SYCP3 | Novus | Cat#: NB300-230 |
| γH2AX | Sigma‒Aldrich | Cat#: 05-636-I |
| RAD51 | Abcam | Cat#: ab133534 |
| Hoechst 33342 | Invitrogen | Cat#: H3570 |
| Alexa Fluor® 594 Donkey Anti-Rabbit IgG (H+L) | Invitrogen | Cat#: A-21207 |
| Alexa Fluor® 488 Donkey Anti-Mouse IgG (H+L) | Invitrogen | Cat#: A-21202 |
| Alexa Fluor® 594 Donkey Anti-Goat IgG (H+L) | Abcam | Cat#: ab150132 |
| Alexa Fluor® 488 Donkey Anti-Rabbit IgG (H+L) | Invitrogen | Cat#: A-21206 |
| Chemicals |  |  |
| Y27632 | Selleck | Cat#: S1049 |
| Ascorbic acid (Vitamin C, Vc) | Sigma | Cat#: A8960 |
| ICI 182,780 | Tocris | Cat#: 1047 |
| Retinoic acid (RA) | Sigma | Cat#: R2625 |
| Critical Commercial Assays |  |  |
| Follicle-stimulating hormone(FSH) ELISA Kit | EastBiopharm | Cat#: CK-E20419 |
| Anti-Mullerian hormone (AMH) ELISA Kit | EastBiopharm | Cat#: CK-E90200 |
| Experimental Models: Organisms/Strains |  |  |
| NOD-SCID mice | Vital River | #406 |
| Albino ICR mice | Vital River | #201 |
| C57BL/6NCrSlc mice | Vital River | #213 |
| β-Actin-GFP mice |  | #RBRC00267 |
| Software and Algorithms |  |  |
| GraphPad Prism software | https://www.graphpad.com | Version 10.2.0 |
| R | https://www.R-project.org/ | Version 4.4 |
| Seurat | https://satijalab.org/seurat/articles/install.html | Version 5.1.0 |
| ImageJ | http://www.imagej.net | Version 1.53i |
